# Proficiency test for rabies serology: A design complying with international standards for a reliable assessment of participating laboratories

**DOI:** 10.1371/journal.pntd.0007824

**Published:** 2019-12-11

**Authors:** Marine Wasniewski, Michel Laurentie, Franca Rizzo, Alexandre Servat, Michel Aubert, Florence Cliquet

**Affiliations:** 1 ANSES, Nancy Laboratory for Rabies and Wildlife, European Union Reference Laboratory for Rabies Serology, European Union Reference Laboratory for Rabies, WHO Collaborating Centre for Research and Management in Zoonoses Control, OIE Reference Laboratory for Rabies, Malzeville, France; 2 ANSES—Platform of Statistical Analysis for proficiency testing and validation of analysis method (PAS), Fougères, France; 3 ANSES (retired) 1088 chemin des Maures, Callian, France; US Department of Agriculture, UNITED STATES

## Abstract

**Background:**

Domestic carnivores can introduce rabies into disease-free countries or areas if they are incubating the disease and transported during the pre-symptomatic period. For pets moved into the European Union, the European Commission decided to establish a system of community approval of laboratories willing to carry out the rabies serological controls to guarantee an effective control system. As the specific institute to coordinate the approval of the laboratories, designated by the European Commission in 2000, our laboratory organizes annual proficiency tests (PT) for laboratories already agreed or willing to be agreed to perform rabies serological controls (by detecting rabies virus neutralizing antibodies only) in the frame of international trade.

**Methodology/Principal findings:**

The assessment criteria of this PT rely on the analysis of the specificity and the intra-laboratory consistency. The approach used to evaluate the degree of laboratory consistency is based on the use of compiled data obtained from previous PT campaigns, and is measured by the quality of a regression model. By using historical data for calculating assigned values and associated standard deviations, instead of values obtained from only one campaign, they became robust without any additional statistical treatment. In the present paper, more than 800 historical values were compiled for each of the regression parameters.

**Conclusions/Significance:**

Since the beginning of these PT schemes in 1999, the overall percentage of failing laboratories remained stable over the years (4.1%) while the number of participants increased to 79 in 2018. This highlighted the robustness and the consistency of the statistical analyses used to assess the laboratory’s performance over the years. The improvements carried out and the consistency of our statistical analyses have resulted in the compliance of the rabies serology PT with the ISO/IEC 17043 and ISO 13528:2015 International Standards.

## Author summary

Proficiency testing (PT) schemes also known as “external quality assessment schemes” are one means to assess the quality and validity of routine measurements. PT schemes are the most common, and perhaps the most important type of inter-laboratory comparisons. As the specific institute to coordinate the approval of the laboratories, designated by the European Commission in 2000, our laboratory organizes annual PT for laboratories already agreed or willing to be agreed to perform rabies serological controls in the frame of international trade. Our PT program is conducted in compliance with the requirements of the ISO/ IEC 17043 International Standards. To enhance the robustness of our statistical analyses, we used an original approach to assess the performance of participants consisting in comparing individual laboratory values with assigned values calculated from compiled PT historical data. Since the beginning of these PT schemes in 1999, the overall percentage of failing laboratories remained stable over the years. The improvements carried out and the consistency of our statistical analyses have resulted in the compliance of the rabies serology PT with the International Standards.

## Introduction

For many years, rabies free territories used to impose a strict quarantine period for the pets coming from infected countries [1]. This period was generally between four and six months depending on the country regulations with the aim of avoiding rabies introduction and therefore to maintain the rabies free status of the country.

Upon arrival in those countries, dogs and cats were placed in quarantine in state establishments under the control of National Veterinary Authorities [2]. This system has proven to play a protective role as, for example in the United Kingdom where 27 cases of rabies were confirmed during the quarantine period, whereas no case of rabies was detected in the country for 22 years (between 1971 and 1993), despite a massive import of pets [1]. Quarantine has thus prevented the introduction or the spread of the rabies virus in the country [2].

However, even if the quarantine confinement proved to be a good method against rabies introduction in rabies free territories, it was unsatisfactory for animal health (physically and psychologically) and for the owner (psychologically and costly) [3].

In 1992, the World Health Organisation (WHO) recommended for the first time a scheme as an alternative measure to quarantine period for the rabies free territories or zones not applying strict quarantine rules. The alternative method combined a rabies vaccination of the pet followed by a serological control to check the effectiveness of the vaccination [4]. These new recommendations were scientifically based on the fact that vaccination against rabies was and is still today the major prophylactic measure to control rabies in animals with the main aim of protecting human health [5]. Indeed it has been shown that cats and dogs are protected against a rabies challenge, provided they have acquired detectable neutralising antibodies (≥ 0.5 IU/mL) whatever the protocol of vaccination used [5]. In 1993, in the same context, the World Organisation for Animal Health (OIE) recommended that after the vaccination and the serological control the animal must wait 3 to 24 months before leaving the country [6]. The waiting period allows to ensure that the rabies antibodies detected are produced in response to the vaccination against rabies and not to the disease itself. Moreover, a directive (92/65/EEC) of the European Commission (EC) provided for an alternative system to quarantine for the entry of certain domestic carnivores into the territory of certain rabies free Member States. This system requires the check on the effectiveness of the vaccination of those animals by titration of rabies antibodies. Between 1993 and 2000, many rabies free countries have alleviated their quarantine measures and adopted a scheme requiring a vaccination followed by a serological control [7, 8]. So, this new scheme, promoted by the WHO, the OIE and the EC, has allowed the free circulation of pets from countries without rabies or where rabies is under control.

For the laboratories willing to carry out the rabies serological analyses of pets entering the EU, the EC decided that it was appropriate to establish a system of community approval of such laboratories to guarantee an effective system to monitor them. The EC decided also that the technical approval of the laboratories should be coordinated by an European Union Reference Laboratory. In March 2000, the European Commission designated our laboratory as the specific “Institute responsible for establishing the criteria necessary for standardising the serological test to monitor the effectiveness of rabies vaccines” (European Decision 2000/258/EC [9]). Among the different duties linked to this mandate, the main task consists in the organisation of rabies proficiency tests (PT) for the EC approval of world laboratories willing to perform the rabies serological controls in the context of the international movements of pets to EU, to assess the performance of laboratories for specific tests. Therefore, our laboratory has organized PT since 1999 and the number of participants has increased continuously. In November 1999, 14 laboratories participated in the proficiency test and in April 2018, 79 participants took part from around the world.

As this PT is mandatory, the participation rules for obtaining, maintaining or failing the European approval being governed by the European Decisions 2000/258/EC and 2010/436/EC [9, 10], and as the number of participants is high and continues to increase over time, it became necessary to prove the robust organization for both the panel preparation and the statistical analysis phases, in order to guarantee a reliable evaluation and objective performance of laboratories.

That is why since 2009, our laboratory has adapted both the structure of the sample panel and the assessment criteria of the participants to comply with the ISO/IEC 17043:2010 International Standard [11]. For the statistical approach, different possibilities are described in the ISO 13528:2015 Standard [12] notably to calculate the assigned values and the standard deviation for proficiency assessment (σPT). In our PT, this latter parameter is only used for assessing the homogeneity of the panel of samples before sending to participants and does not interfere in the evaluation of the laboratory’s performance. This paper presents the description of the annual rabies serology proficiency test and the last adaptations performed to comply with the international Standards as well as the statistical approach used for analyzing participants’ data.

## Material and method

The rabies serology proficiency test procedure has been elaborated in 1999 with laboratories involved in rabies serology and then updated through regular meetings organised within the rabies serology laboratory network and with the European Commission. This procedure describes the overall organisation of the annual proficiency test for rabies serology. It is based on the following documents:

- the OIE guidelines for the evaluation of veterinary laboratory capability to conduct diagnostic tests for infectious diseases: Laboratory Proficiency Testing: (http://www.oie.int/en/our-scientific-expertise/reference-laboratories/proficiency-testing/).
- the Council Decision 2000/258/EC of 20 March 2000 (http://eur-lex.europa.eu/legal-content/EN/TXT/PDF/?uri=CELEX:02000D0258-20080903&qid=1496305585562&from=EN).
- and the Regulation (EU) No 576/2013 of the European Parliament and of the Council of 12 June 2013 on the non-commercial movement of pet animals and repealing Regulation (EC) No 998/2003 Text with EEA (Europe Economic Area) relevance.

**Fig 1:**
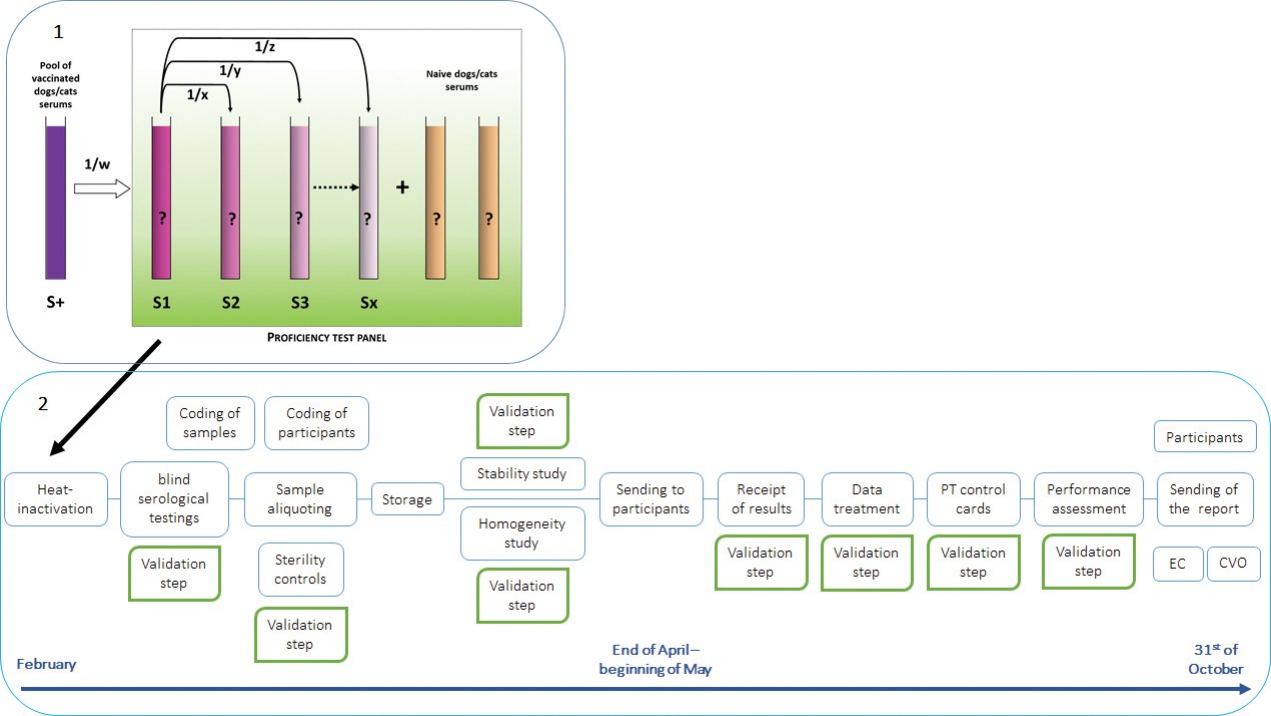
1- Composition and preparation of the proficiency test panel for assessing the performances of the laboratories. The panel contains two or more naïve sera from unvaccinated pets and eight or nine samples from vaccinated dogs/cats. These sera correspond to a high titre serum S1 (obtained from a pool of sera) and several dilutions of this serum (sera S2 to Sx). 2- **Flow chart presenting the main steps of the rabies serology PT scheme organisation once samples have been prepared. EC: European Commission, CVO: Chief Veterinary Officer.**

The main steps of the rabies serology PT scheme organisation are presented in Fig 1.

### Panel composition

This panel is as far as possible representative in terms of matrix and rabies antibody concentrations of the type of field samples analysed by the testing laboratories. Each year, sample panels are processed and provided by our laboratory to participating laboratories. Panels contain some sera of dog/cat origin taken from unvaccinated animals or animals vaccinated against rabies by the parenteral route using vaccines recognised by the WHO. Generally, the panel contains two or more naïve sera from unvaccinated pets and eight or nine samples from vaccinated dogs/cats. These sera correspond to a high titre serum called S1 (obtained from the pool of sera) and several dilutions of this serum in a sterile buffered saline solution as shown in Fig 1. Dilution factors are odd (therefore unpredictable) and every dilution is made directly from the high titre sample S1 to avoid the repetition of any possible dilution error along the series. Before aliquoting, all sera are heat-inactivated and the panel is blindly titrated several times by different operators to assess the consistency of the dilution factors and to make sure there was no problem of cytotoxicity. This ensures the unequivocal status of the test material. Then, the different sera constituting the panel are distributed in individual vials with a sufficient volume to perform at least three independent tests as required in this PT. All the vials are stored at –20°C. Moreover as mitigation to prevent collusion between laboratories, one or several mock samples are included in the panel. Therefore the results obtained for the latter were never analysed.

Each sample of the panel is coded as well as the participating laboratories so that in one hand every participating laboratory tests blindly the panel of sera and in the other hand the statistical analyses are also performed blindly by our laboratory. These codes are randomly drawn.

### Homogeneity and stability

#### Homogeneity

Each item of 10 randomly selected panels is tested in duplicate to assess the homogeneity in accordance with the specifications of the ISO 13528 International Standard [12]. For each item of the panel, the measurements are made under repeatability conditions. From these measurements, the general average, the within-sample standard deviation and the between-sample standard deviation are calculated. Then, the between-sample standard deviation (s_*s*_) obtained for each item is compared with the standard deviation for proficiency assessment (σPT). The proficiency test item is considered to be adequately homogenous if (s_*s*_) ≤ 0.3 σPT such as described in the ISO 13528 Standard [12].

The σPT is calculated from historical data. Practically, this value is calculated from the set of the logarithms of the dilution 50's (logD50 values; i.e. the endpoint dilution where 50% of the wells showed the presence of virus–see section Methods used) of each item belonging to the dilution range of the panel for all the participants of the 5 previous campaigns (representing more than 7400 values). σPT corresponds to the square root of the mean of the variances calculated for each item.

#### Stability

The stability of the items is assessed in conditions (time and temperature) mimicking a transport, by using an alternative method to the one described in the ISO/IEC 13528 Standard [12]. One prepared panel is selected randomly and the sera belonging to the dilution range panel as well as the naïve samples are used to evaluate the stability. These sera are titrated by using the Fluorescent Antibody Virus Neutralisation test (FAVN test–see section Method used) at different times: D0, D7, D14 and around D50 (considering the 45 day period allowed to send the official results). From D0 to D14, the samples are stored in a parcel at room temperature, mimicking a long transport or a problem during the transport (e.g. panel blocked at customs). Then the samples are stored at -20°C mimicking a receipt and storage by the participating laboratory.

For the naïve samples, the stability is assessed by checking that the value obtained for each time (D0, D7, D14 and post freezing) is always less than 0.5 IU/mL and close to 0 IU/mL.

Regarding the sera belonging to the dilution range panel, for each test, a simple linear regression is performed between experimental and theoretical logD50 values. The theoretical logD50 are deduced from the value found for the undiluted sera (serum S1) and by using the scale of dilution of sera. As the experimental results are a function of the theoretical values, the comparison is made according to a simple linear regression (y = bx + a), with b and a representing the theoretical and experimental values respectively.

For each tested day, the values obtained from the slope “b” and the y-intercept “a” are compared respectively to the historical mean values +/- 1.96 historical Standard Deviation (SD) value obtained a priori and a posteriori, i.e. before and after the current campaign. The value obtained for the coefficient of determination (R^2^) is compared to the historical 90^th^ percentile value (P90) calculated a priori and a posteriori as well.

If, for the 4 regression curves obtained at D0, D7, D14 and post-freezing, the values of “a” and “b” are in the range of the historical mean +/-1.96 SD and the R^2^ value is above P90, therefore the items are declared stable for the proficiency test.

### Methods used

For the titration of sample panels, participating laboratories shall use the seroneutralization technique(s) recommended in the OIE Manual of Diagnostic Tests and Vaccines for Terrestrial Animals [13],i.e. the FAVN (Fluorescent Antibody Virus Neutralization) test [14, 15] or the original RFFIT (Rapid Fluorescent Focus Inhibition Test) practised on Labtek chamber slides [16]. There must be no variation in performing either of these methodologies whether they are applied to the proficiency test or for usual serum titration. Higher dilutions additional to the usual protocols that may be required for providing precise values for some sera of the panel are not considered as protocol variations. For each laboratory, every sample should be tested in three independent tests.

The laboratories shall include a reference serum in each test. It should be preferably the OIE reference serum of dog origin diluted to 0.5 IU/mL [17] but the laboratories could use also the WHO rabies human immunoglobulin preparation [18] as reference serum or an internal preparation previously validated against one of the two international reference sera (i.e. OIE or WHO).

### Panel testing

Participating laboratories have to run three independent titrations of the test panel by using their current own protocol. Results must be expressed in precise logD50 values and IU/mL values with two decimals only and not expressed as open intervals.

Participating laboratories should return results within 45 days of test panel receipt by using a web interface to secure data.

After validation of data, participants have the possibility to modify data if needed until the settled deadline. In order to assume the results, the participant must print and sign the paper form and then send it to the PT provider. The PT provider acknowledges receipt of the results after receipt of this signed form.

### Statistical analysis of results—Pass/fail criteria

Results obtained by participating laboratories must be expressed in precise logD50 values and IU/mL values with two decimals only. Two criteria, which are the specificity and the intra-laboratory consistency, are assessed by using appropriate statistical methods in order to interpret the experimental values obtained by the participating laboratories.

#### Specificity

The first criterion is the specificity, which corresponds to the ability to identify serum from naïve dogs/cats. Each laboratory should determine titres of naïve dogs and cats sera as being strictly below the value of 0.5 IU/mL, which is the threshold of protection fixed by the international authorities (WHO and OIE).

Any false positive result is a severe fail criterion.

Obtaining from naïve animal sera values lower than 0.5 IU/mL but very close to this threshold is not a fail criterion but may indicate a lack of specificity.

#### Intra-laboratory consistency

The intra-laboratory consistency is used to determine the trueness of each participating laboratory. This criterion is evaluated by comparing the logD50’s given by the laboratory under study (experimental D50’s) with the values that this laboratory should have found (expected D50’s). Expected D50's for every laboratory under test are evaluated by using the scale of dilutions of sera and the value found by the laboratory for the serum S1 (coded differently according to the laboratories). Ideally, all experimental results should be equal to the respective expected results. In mathematical terms, y (the experimental results) is a function of x (the expected values) according to a linear regression y = bx + a where b and a are the slope and the y-intercept respectively. The degree of consistency is measured by the quality of the regression i.e., R^2^, b and a parameters. The calculation of these three parameters is done by using the values obtained by each participating laboratory for the three gathered runs.

This graphic distribution of data is first assessed for each participating laboratory to detect the presence of any atypical results. Whether the data given by a participating laboratory are considered as atypical (i.e. non-linear distribution of results, significant inverse correlation…), the results cannot be analyzed by the statistical model. In such case, the laboratory fails to the proficiency test and its results are not considered in the analysis of laboratory’s performances.

The determination of assigned values is done as follows: assigned values are established by using the cumulated values obtained in the previous PTs (from 2006 until now). As the previous PTs have been performed for many years, mean targets for b, a and R^2^ are considered as robust. Consequently, these three values are considered as assigned values. For the same reason, the estimation of the standard deviations (SD) (1.96 SD corresponds to a 95% confidence interval (CI) and 2.575 SD corresponds to a 99% CI) for the a and b parameters as well as P90 and P98 for R^2^ are considered as strong estimators.

Moreover, from a statistical point of view, writing a +/- (1.96 or 2.575) SD or b +/- (1.96 or 2.575) SD is equivalent to use a confidence interval (CI) of parameter and allows to estimate if the value is close to the assigned value.

To obtain a robust mean for the values of slope “b”, y-intercept “a” and R^2^ and their respective SD and P90 and P98, the historic data of the previous PTs were used. Indeed these values were calculated from the historical data from all laboratories obtained since the 2006 PT campaign and the current campaign but excluding the values obtained in case of atypical results.

The analyses of each component of the linear regression curve are done as follows:

The values of the slope (b) and of the y-intercept (a) obtained for the three gathered runs by each participant are compared to the mean value of b and a +/- 1.96 and 2.575 SD, corresponding to a confidence interval of 95% (risk α = 5%) and 99% (risk α = 1%), respectively. The distribution of the b and a values obtained by each participating laboratory, are reported on two independent figures, and three areas are defined in each figure:

- The “expected” area where the values obtained should be less than the mean +/-1.96 SD.
- The “warning” area where the values obtained should be equal to the mean +/-1.96 SD and less than the mean +/- 2.575 SD.
- The “action” area where the values obtained should be equal to or above the mean +/-2.575 SD.

The limit values of R^2^ are also determined from historical data and those of the current campaign.

The distribution of the coefficients of determination is characteristic of a one-sided distribution and limits are fixed to the 90th (P90) and 98th (P98) percentiles of historical values. These limits amount to a risk level (1–2α), i.e. 90% and 98%, to correspond to the risks applied to the slope b and the y-intercept a.

The distribution of the R^2^ values obtained by each participating laboratory is reported on one figure where three areas are defined:

- The expected area where the values obtained should be above P90.
- The warning area where the values obtained should be equal to P90 and more than P98.
- The action area where the values obtained should be equal to or below P98.

The Pass/Fail criteria were set according to the following rules:

For the pass criteria,

If the coefficient of determination (R^2^) value is in the expected area, and if

the y-intercept (a) value is in the expected area, and if

the slope (b) is in the expected area, the laboratory is considered as successful to the annual PT.

For the moderate fail criteria resulting in a “close examination” of each step of the serological test,

If one of three values (coefficient of determination (R^2^), slope (b) or why intercept (a)) is in the warning area, the laboratory is invited to consider the different steps of the serological test to identify the source of the deviation.

For the severe fail criteria resulting in the failure to the annual proficiency test,

If one of the three values (coefficient of determination (R^2^), slope (b) or y-intercept (a)) is in the action area, the participating laboratory is considered as unsuccessful to the annual PT.

## Results

### Historical data: Assigned values

**Fig 2:**
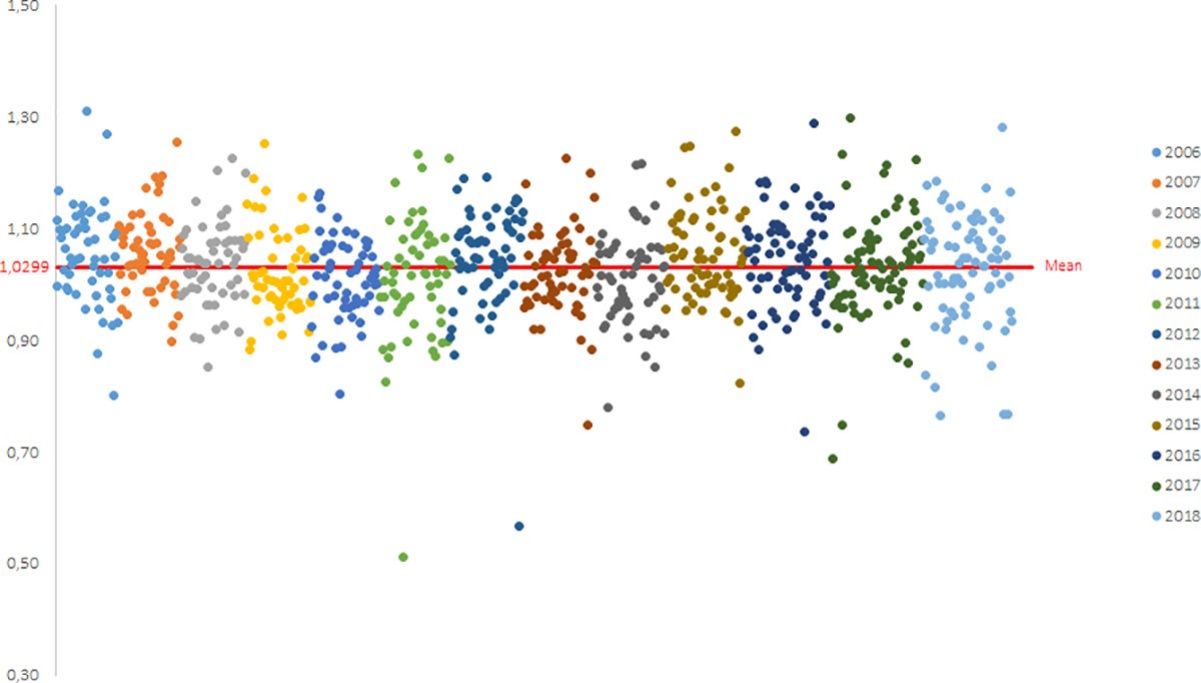
Calculation of the historical mean of the slope b (one dot represents one participant): distribution of the slope b values obtained by participants from PTs between 2006 and 2018.

**Fig 3:**
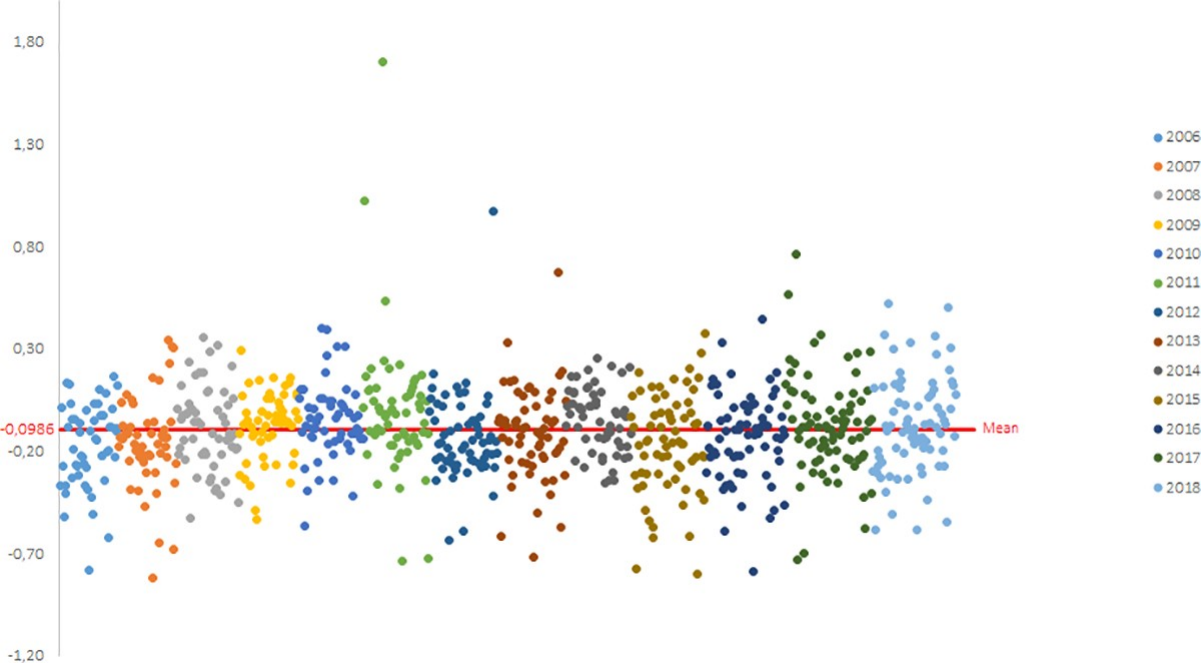
Calculation of the historical mean of the y-intercept a (one dot represents one participant): distribution of the y-intercept a values obtained by participants from PTs between 2006 and 2018.

**Fig 4:**
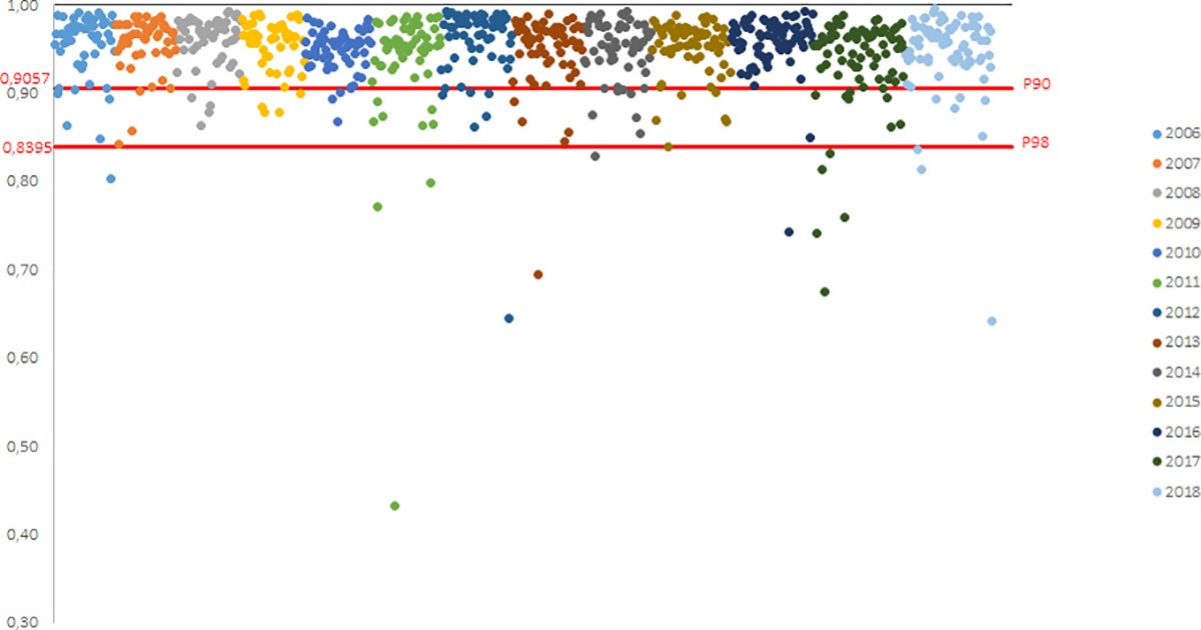
Calculation of the historical P90 and P98 values of the R^2^ values (one dot represents one participant): distribution of the R^2^ values obtained by participants from PTs between 2006 and 2018.

Between 2006 and 2018, 813 laboratory values were obtained for each of the three parameters (the slope b, the y-intercept a and the R^2^). The distribution of the values obtained for each campaign between 2006 and 2018 are shown on the Figs 2, 3 and 4 for the slope b, the y-intercept a and the R^2^, respectively.

According to these cumulative data, the mean value for the slope b is equal to 1.0303 (n = 813) with a standard deviation of 0.1027 and the mean value for the y-intercept a is equal to -0.1012 (n = 813) with a standard deviation of 0.2266. For the R^2^, the P90 value is equal to 0.9055 (n = 813) and the P98 value is equal to 0.8356 (n = 813).

### Examples of results obtained by the laboratories

**Fig 5:**
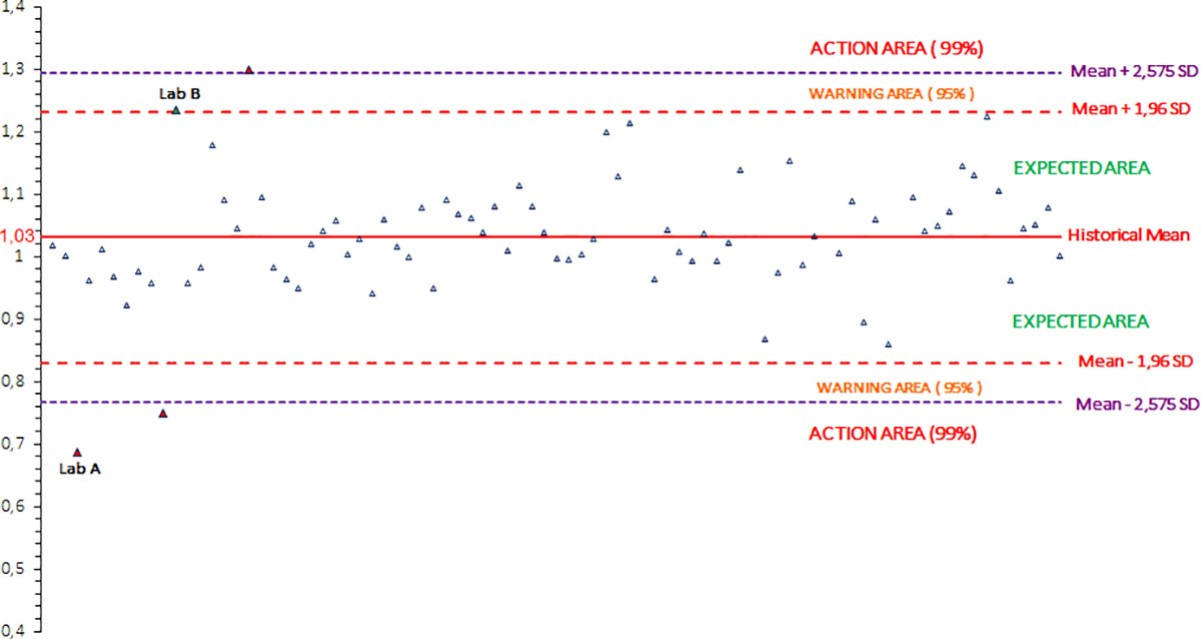
Example of the distribution of the b values obtained by each participating laboratory for the three gathered runs for one rabies serology proficiency test campaign (one dot represents one participant).

**Fig 6:**
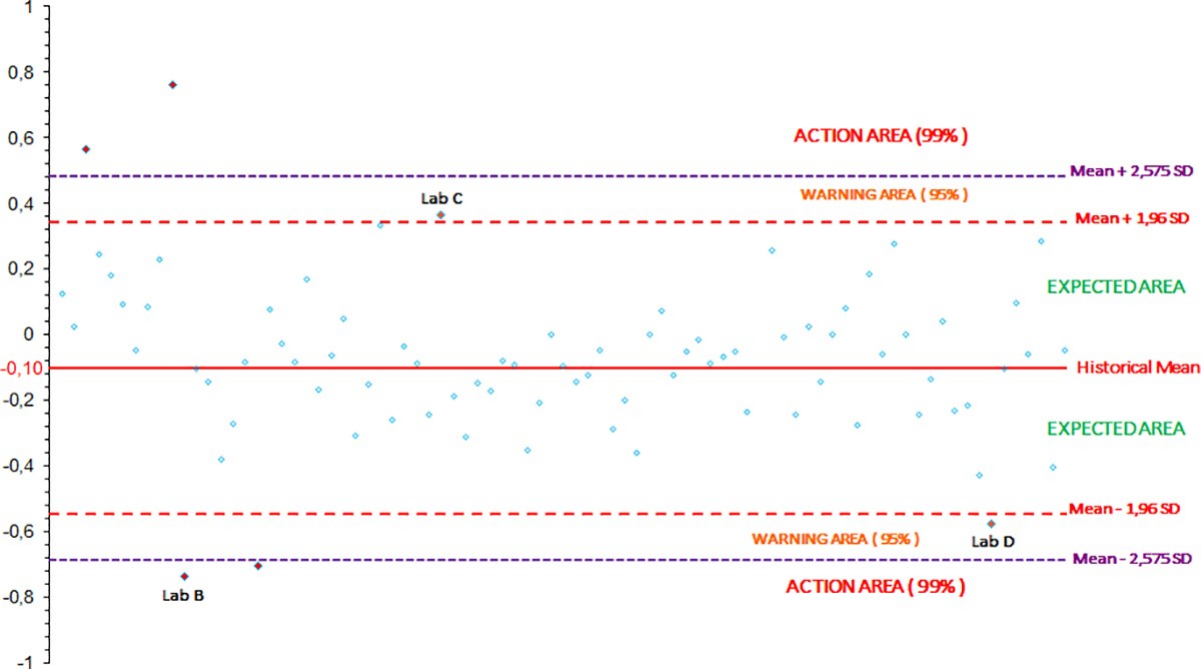
Example of the distribution of the a values obtained by each participating laboratory for the three gathered runs for one rabies serology proficiency test campaign (one dot represents one participant).

**Fig 7:**
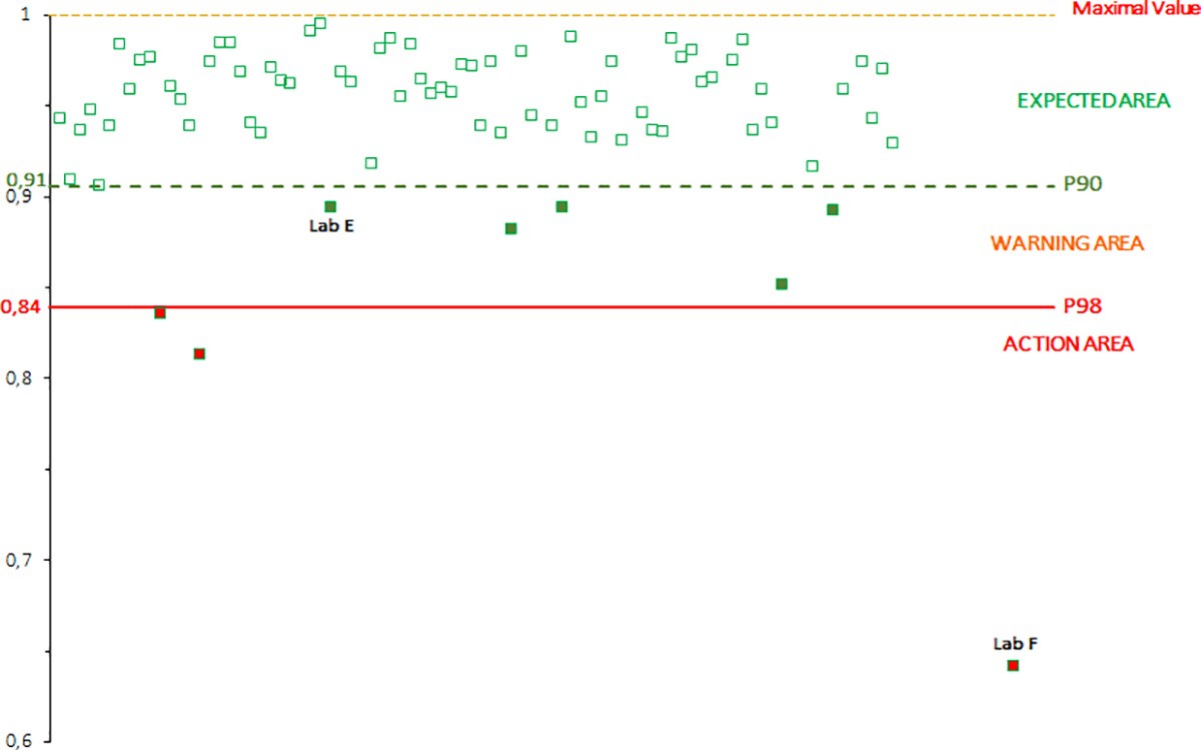
Example of the distribution of the R^2^ values obtained by each participating laboratory for the three gathered runs for one rabies serology proficiency test campaign (one dot represents one participant).

The Figs 5, 6 and 7 record the data obtained by the participating laboratories during a PT campaign for the slope b, the y-intercept a and the R^2^, respectively. Several laboratories could be highlighted, as examples, regarding their more or less unsatisfactory performances.

- For laboratory A, the value of the slope (around 0.69) was in the action area, i.e. below the mean -2.575 SD limit (equal to 0.77).
- For laboratory B, the value of the slope (1.24) was in the warning area, i.e. between the mean +1.96 SD (equal to 1.23) and the mean +2.575 SD (equal to 1.30). Moreover, the value of the y-intercept (around -0.73) was in the action area, below the mean -2.575 SD limit (equal to -0.68). This significant y-intercept value corresponded to a very high overestimation of the dilution factors (a dilution of more than 1/5.4 when the expected dilution was 1/1).
- For laboratory C, the value of the y-intercept (around 0.37) was in the warning area, i.e. between the mean +1.96 SD (equal to 0.34) and the mean +2.575 SD (equal to 0.48). This corresponded to a high underestimation of the dilution factors (a dilution of 1/0.4 when the expected dilution was 1/1).
- For laboratory D, the value of the y-intercept (around -0.58) was in the warning area, between the mean -1.96 SD (equal to -0.55) and the mean -2.575 SD (equal to -0.68). This corresponded to a high overestimation of the dilution factors (a dilution of 1/3.8 when the expected dilution was 1/1).
- For laboratory E, the value of the coefficient of determination (R^2^) (around 0.89) was in the warning area, i.e. between the P90 value (equal to 0.91) and the P98 value (equal to 0.84).
- For laboratory F, the value of the coefficient of determination (R^2^) (around 0.64) was in the action area, below the P98 value (equal to 0.84).

### Analysis, reporting and authorisation

On the basis of the statistical analysis and the above mentioned criteria, our laboratory (PT provider) produces a report including raw data, individual performance of all participating coded laboratories, statistical analysis and the interpretation of the final results (pass/close examination/fail) of the annual proficiency test. Laboratories are designated by an individual code that could be different from a PT to another. Every laboratory is informed only of its individual code for this run of proficiency test; the other laboratory codes are not divulged.

**Fig 8:**
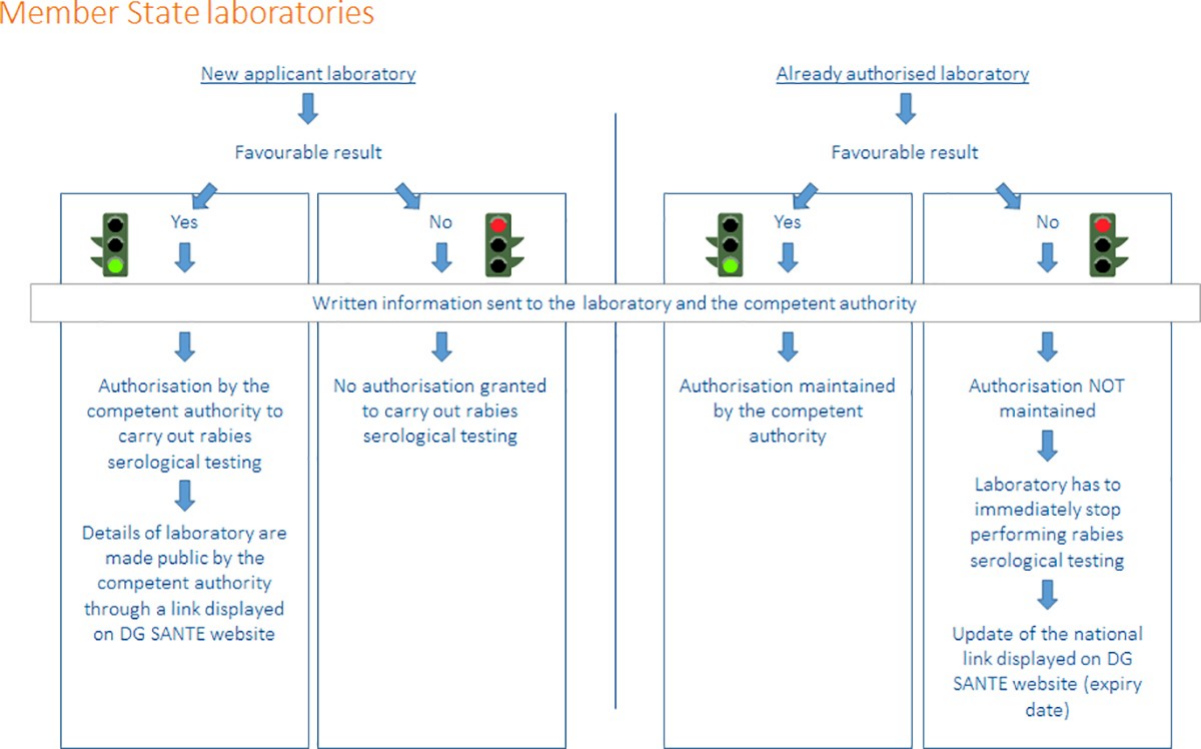
The process for obtaining, maintaining or losing its European approval for the rabies serological testing of pets for a Member State laboratory.

**Fig 9:**
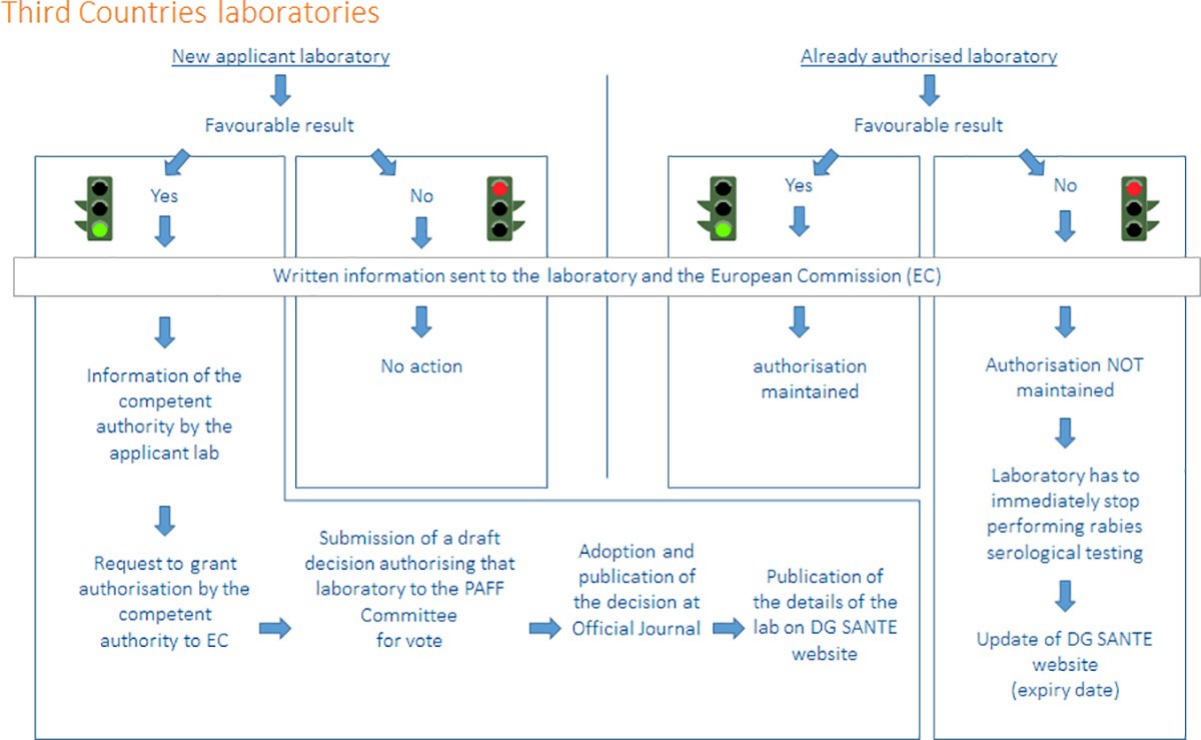
The process for obtaining, maintaining or losing its European approval for the rabies serological testing of pets for a Third country laboratory. PAFF Committee: Standing Committee on Plants, Animals, Food and Feed.

The technical analysis report issued by our laboratory is sent electronically to each participating laboratory. As shown on the Figs 8 and 9, a letter indicating the code of each respective laboratory and the result (favourable or failure) is sent to the laboratories and to the competent authority of the Member State where the laboratory is located or to the European Commission if the laboratory belongs to a non EU country, at the latest by 31 October of the same year, according to the Decision 2010/436/EC [10].

A moderate fail criteria resulting in a “close examination” is only notified in the report. In such case, the PT provider advises the laboratory to undertake corrective measures to improve the quality of the results. A close examination does not entail the appraisal status of the laboratory.

The letter also informs the competent authority of the Member States that in case of a favourable result it may authorise or maintain the authorisation of this laboratory to carry out the serological tests to monitor the effectiveness of rabies vaccination in dogs, cats or ferrets (Fig 8). If it decides so, the details of that laboratory should be made public at a national dedicated website whose link is displayed (http://ec.europa.eu/food/animals/pet-movement/approved-labs_en).

In case of failure of an authorised laboratory, the authorisation granted to that laboratory by the competent authority should not be maintained and the laboratory has to immediately stop performing the rabies serological tests to monitor the effectiveness of rabies vaccination in dogs, cats and ferrets travelling under EC regulations only according to the Decision 2010/436/EC [10]. The competent authority should immediately update its website by adding an expiry date to inform the public of the failure, i.e. the laboratory has no longer EC approval for testing those pets travelling under EC regulations.

As shown on Fig 9, in case of a favourable result for an applicant laboratory located in non-EU country, the authorisation to carry out the serological tests to monitor the effectiveness of rabies vaccination in dogs, cats or ferrets may be granted to that laboratory if the competent authority of the non-EU country where the laboratory is located, requests so to the European Commission. In that case, the European Commission would prepare and submit for an opinion of Member States a draft Commission Decision authorising that laboratory. When that Decision is adopted by the European Commission and then published at the Official Journal of the European Union, the details of the laboratory are displayed at the following Commission website: http://ec.europa.eu/food/animals/pet-movement/approved-labs_en. In case of a favourable result for an authorised laboratory, the authorisation granted to the laboratory is maintained. In case of failure of an authorised laboratory, the authorisation granted to the laboratory should not be maintained and the laboratory has to immediately stop performing the rabies serological tests to monitor the effectiveness of rabies vaccination in dogs, cats or ferrets travelling under EC regulations only according to the Decision 2010/436/EC [10] like for the Member State laboratories. The European Commission should immediately inform the competent authority and the laboratory and update the aforementioned Commission website.

## Discussion

Proficiency testing schemes also known as “external quality assessment (EQA) schemes” are one means to assess the quality and validity of routine measurements. PT schemes are the most common, and perhaps the most important type of inter-laboratory comparisons. PT schemes used in biology could be organized and evaluated in many different ways depending particularly on the objective of the assay, on the techniques used, the nature of the analyte tested (antigen or antibody).

In this PT program, there is no particular statistical requirement for the minimum number of participants because the methodology for preparing the serum panels did not change since the start in 1999. The same year, international rabies experts discussed on the objective of this PT, the pass fail criteria and the method for analyzing the data provided by the laboratories. The objective of the PT is to evaluate the technical performances of laboratories to carry out the serological tests to monitor the effectiveness of the anti-rabies vaccination of dogs, cats and ferrets, i.e. to qualify vaccinated animals in the context of alleviated measures to rabies quarantine. In this context, the specificity criterion is of outstanding importance to avoid the movement of a false positive animal, i.e. an animal that could be not vaccinated or not protected, from a territory where rabies is endemic. The second criteria is to assess the intra laboratory consistency. Despite improvements in standardization of seroneutralization techniques [14, 19], these methods remain more variable than binding assays (such as ELISAs) [20], hence the statistical analysis of results can hardly been simply performed by comparing values given by the laboratories with assigned values of the provider for each serum tested. Using the 3 gathered logD50 values of the highest IU/mL serum (S1) determined by each laboratory and checking all dilution factors from this serum are found as statistically reliable, is a consistent way to assess the individual technical performance of the test in the dilution range of the test. This method allows to calculate for each participant the slope b, the y-intercept a and the coefficient of determination R^2^ of the regression analysis between values found by the laboratory and values that the laboratory should have found according to the dilution factors. These individual three values are compared to three assigned values of b, a and R^2^ which are compiled from historical data from previous campaigns and including the current campaign. By using the historical data for calculating the assigned values and their associated SD instead of only the values obtained during the current campaign, they became robust values without any additional statistical treatment (i.e. no need to use robust method such as the algorithm A (ISO 13528:2015 [12]) or Hampel-/Q-method (ISO/TS 20612 [21])). Nevertheless, the proposed statistics are in agreement with the statistical approach described in the ISO 13528 International Standard [12]. Indeed, in our PT scheme, the assigned values are determined from the results of participants on the whole PT performed from many years. This approach is very close to one described in the ISO 13528:2015 Standard [12] suggesting a consensus value from participant results obtained from the current PT as assigned value.

A study [19] comparing the performances of FAVN test and RFFIT demonstrated that no difference in the sensitivity or specificity was observed for both methods (around 86% of the participating laboratories are using the FAVN test [15]). We found also that there was no difference in the results provided by participants linked to the methods used in our PT schemes (either the FAVN test or the RFFIT). Therefore, the calculation of the consensus value is not biased by using the values obtained by both methods and the method used by the laboratory to titrate the PT samples does not affect the assessment of the laboratory’s performance.

Since the beginning of these PT schemes in 1999, the number of failed laboratories (between 0 and 9 laboratories annually, irrespective of the test used (RFFIT or FAVN test), representing an overall percentage of 4.1% of failures) has remained stable over the years while the number of laboratories increased from 14 in 1999 to reach 79 in 2018 (S1 Fig). This highlighted the robustness and the consistency of the statistical analyses used to assess the laboratory’s performance over the years. After discussing with the relevant laboratories and analyzing results, we were able to establish the main reasons resulting in a laboratory failure. The latter could result from problems linked to the staff (e.g. recent change in staff, lengthy absence…), to the virus (e.g. non-appropriate working dilution of the virus resulting in a lower or upper dose compared to the one required for the test,…), to the cell line (e.g. mycoplasma contamination, loss of sensitivity due to too many passages, non-respect of the concentration of cells added in the wells,…), to the absence of test validation by using control cards for the positive, negative control and the virus back titration, to the reading (e.g. artefacts could be considered as positive cells, incorrect settings of the fluorescent microscope,…) or to the equipment (e.g. pipettes not under metrological control delivering an incorrect volume,…).

As EURL for rabies serology, we shall provide a technical and scientific assistance to the laboratories. This could be done by the organization of training courses, e-mail and technical protocol exchanges, sending of critical reagents (CVS-11 virus, positive and negative reference serum) and/or training material (training panel or blind serum samples). We also provide this assistance to new laboratories willing to be approved for rabies serology, to help them to properly implement the seroneutralization test in their premises.

With the results obtained by participating laboratories in this PT scheme over the years, and the sharing of SOPs carried out in different laboratories, we have been able in collaboration with the network of approved laboratories to refine the rabies seroneutralisation techniques by identifying the key technical parameters to strictly consider when performing rabies antibody titrations. By this way, harmonized and detailed SOPs have been published by OIE and WHO [13, 22] for the attention of laboratories already approved or willing to be approved by the EC for rabies serology.

Some limitations could be mentioned. First, it is important to keep in mind that the PT corresponds to a snapshot of the situation of the laboratory at a given time. That is why, in addition to participation in the PT scheme, laboratories should have internal controls to validate their tests regularly and therefore ensure the quality of results all the time. Another limitation is linked to the matrix (serum diluted in sterile PBS) which does not correspond exactly to the samples routinely received in the laboratories as the latter are not diluted in PBS. In the past, we faced up to the need of large volumes of serum samples to provide for organizing the rabies serology PT. We also faced up to the problem of the availability of large quantity of serum samples obtained from unvaccinated domestic animals. Therefore, as an alternative method, we decided to dilute serum samples in sterile PBS. We have previously compared results with both dilutions of sera in naive serum and those in sterile PBS and no difference was reported either in the titres obtained or in the reading step. Furthermore, as the welfare of the animals is concerned, it is much more acceptable to use sterile PBS instead of blood collected from unvaccinated domestic animals for diluting serum samples in the frame of a PT.

Additionally, we have identified some critical points linked to our organization of PT, as they could potentially have an impact on the evaluation of the performance of the participating laboratories. In order to minimize their impact, some mitigations have been implemented to detect and control them. As an ultimate goal for increasing confidence that our PT program is being operated consistently and competently, this latter is in compliance with the requirements of the ISO/ IEC 17043 International Standards [11]. To fit these requirements, the confidentiality is preserved and the risk of collusion between the participating laboratories is avoided by including one (or more) mock sample. Moreover, a worst case has been carried out for the stability study for mimicking the worst conditions of transport. Indeed a panel of samples was stored at 40°C during 50 days and it was tested at different days D0, D7, D21 and D50. The stability was declared satisfactory for all samples.

Opportunities and continuous improvement are seeked. As an example, a satisfaction survey is also sent after the technical report to improve the process through the proposals made by the participating laboratories. Its analysis has resulted in the set-up of an electronic form to return results of the test panel by using a web interface to secure data, the detailed description in the report of the pass/fail criteria to evaluate laboratory performances and the individual electronic sending of the report through a secure web platform. Information sheets are sent with the registration form and with the panel of samples for providing detailed documented instructions to participants and the different deadlines to meet. An internal advisory group has been set up gathering technical experts in rabies serology as well as in statistics and quality management to guarantee impartial and objective decisions. The main tasks of this advisory group were the validation of the draft schedule of the proficiency tests as well as the approval of the technical draft report. Its opinion could also be requested on any questions related to the rabies serology proficiency test. And finally, the consistency of the results of the current PT scheme was checked through control cards, established for the y-intercept a and the slope b. Indeed, for each parameter, the limits of the control card are set using the average of historical values +/- 1.96 SD. The historical values have been obtained since 2006 (including the current campaign), excluding the values for which an atypical profile has been detected (the number of atypical results has varied between 0 and 1 per campaign since 2006). The overall results of all the laboratories for each current annual campaign are then compiled to determine the global average of the y-intercept a and the slope b, excluding values for which an atypical profile has been detected. If these means are within the limits of the control card, the campaign is validated and the individual performance of each participating laboratory (S2 Fig) can be evaluated. If one of the 2 values is out of the control card, an investigation will be done to find the origin and a decision will be made for the assessment of the performances of participating laboratories after consulting the advisory group.

The main issue of this PT scheme is to assess annually the performances of laboratories involved in rabies serology so that they obtain, maintain or lose their annual EC agreement for rabies serological titrations for domestic carnivores. This scheme also provided participants with regular, objective and independent assessment of the quality of their “routine” work, feedback that could lead to the improvement of the technical work and comparative information about the performance of the seroneutralisation tests. Furthermore, laboratories approved by the EC and which are accredited according to the ISO/IEC 17025 International Standard [23], require, among other obligations, an independent inter-laboratory assessment for complying with their respective QA systems and for assuring the validity of their test results. During a survey done in 2017 within the serology network, among 69 approved laboratories contacted, 22 of 33 which responded to the survey (67%) declared being accredited to ISO/IEC 17025 [24].

For the European Commission, this PT scheme provides an overview of the quality of these specific analyses performed worldwide and guarantees the rabies serological results given by the approved laboratories in the frame of international trade and therefore preserves the free rabies status of countries. Since the implementation of rabies serological controls as replacement of quarantine measures, no imported rabies case of domestic carnivore complying with the EC requirements (as defined in the EU regulation 576/2013) was reported through international trades, within and beyond the EU.

The majority of participating laboratories has achieved satisfactory results at each annual proficiency test session since the beginning, indeed between November 1999 and April 2018, the average failure rate is 4.10%. The improvements carried out and the consistency of our statistical analyses have resulted in the compliance of the rabies serology proficiency tests with the International Standards and therefore they have allowed to obtain the ISO/IEC 17043 accreditation from our national accreditation body in May 2017. This accreditation is annually confirmed and allows to validate our process of organization of the PTs (including processes for sample preparation and validation, shipment…), our statistical model to evaluate the performance of participants and our management system linked to the PT organization.

## Supporting information

S1 Fig Success and failure of participating laboratories for the rabies serology proficiency test since 1999. (PDF)

S2 Fig Control cards for the slope "b" and the y-intercept "a" for the three gathered runs for validating each rabies serology proficiency test campaign. (PDF)
