# Supplementary figures and images for: Proficiency test for rabies serology: A design complying with international standards for a reliable assessment of participating laboratories
Source: PLoS Negl Trop Dis. 2019 Dec 11;13(12):e0007824. doi: 10.1371/journal.pntd.0007824 (PMC6905528; doi:10.1371/journal.pntd.0007824)

# Success and failure of participating laboratories for the rabies serology PT since 1999

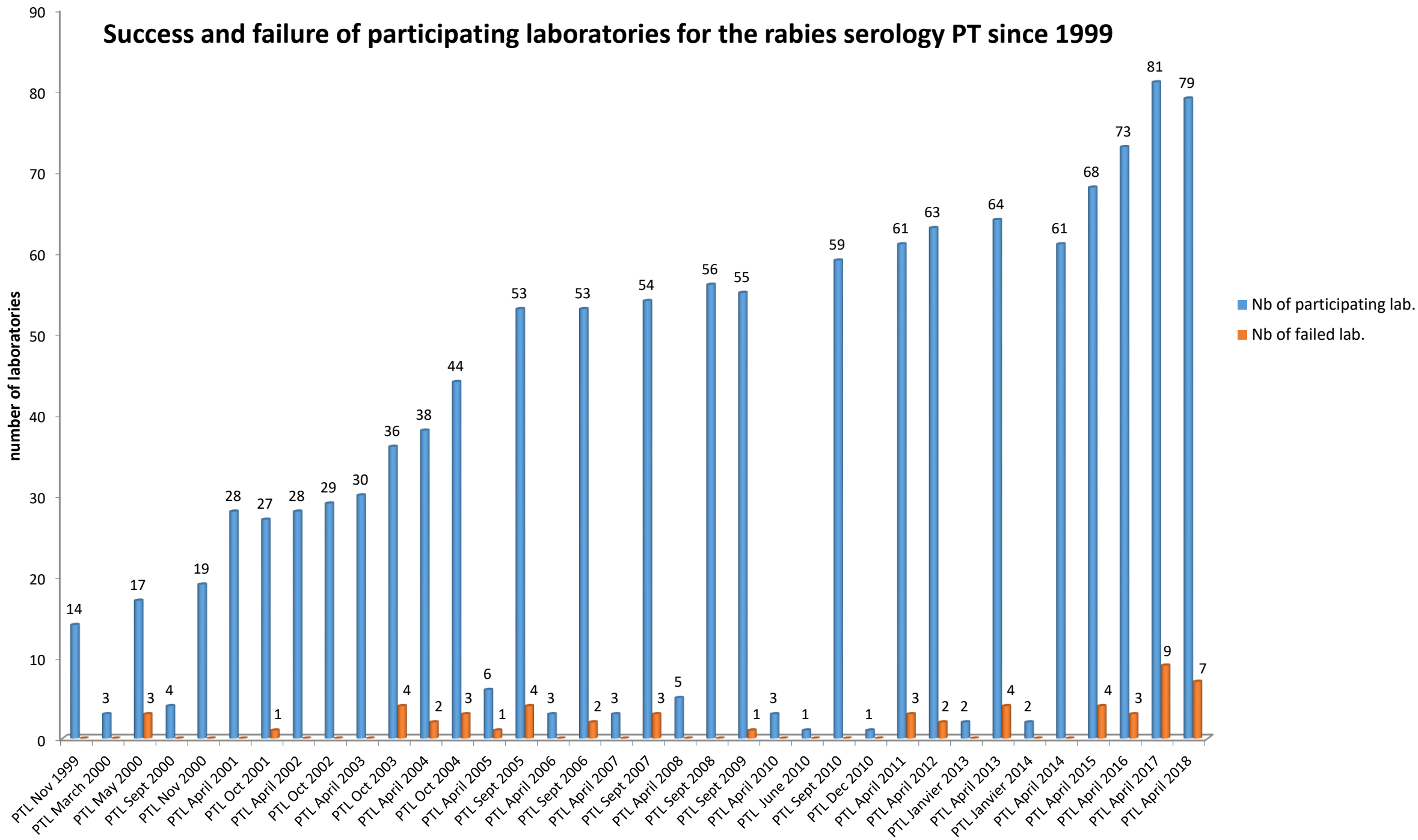

Supplement: S1 Fig — (PDF) [file pntd.0007824.s001.pdf]
