# Supplementary material for: Proficiency test for rabies serology: A design complying with international standards for a reliable assessment of participating laboratories
Source: PLoS Negl Trop Dis. 2019 Dec 11;13(12):e0007824. doi: 10.1371/journal.pntd.0007824 (PMC6905528; doi:10.1371/journal.pntd.0007824)
Supplement: S2 Fig — (PDF) [file pntd.0007824.s002.pdf]

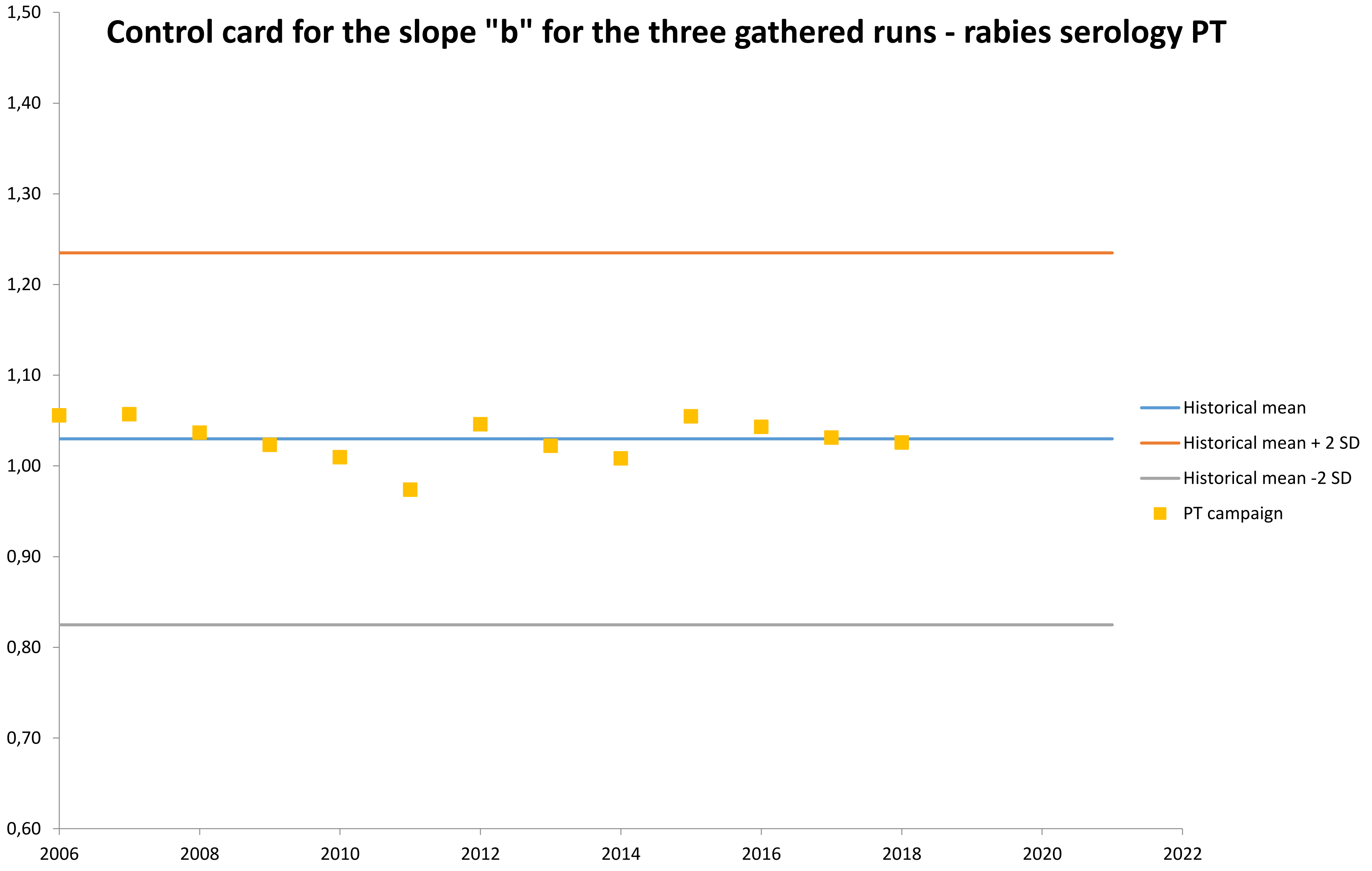
Control card for the slope "b" for the three gathered runs - rabies serology PT
1,50
1,40
1,30
1,20
1,10
1,00
0,90
0,80
0,70
0,60
2006
2008
2010
2012
2014
2016
2018
2020
2022
Historical mean
Historical mean + 2 SD
Historical mean -2 SD
PT campaign

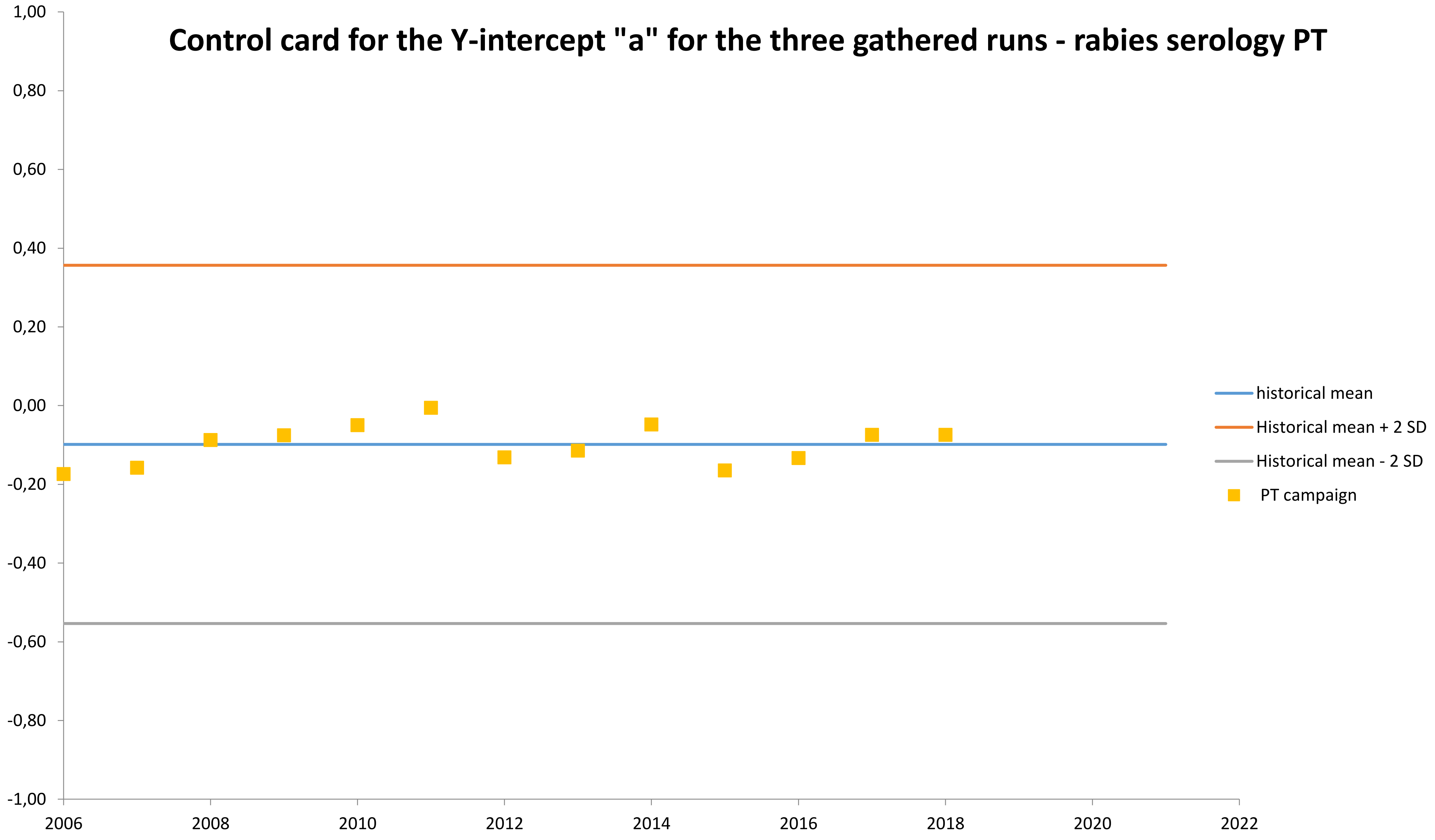

Control card for the Y-intercept "a" for the three gathered runs - rabies serology PT
1,00
0,80
0,60
0,40
0,20
0,00
-0,20
-0,40
-0,60
-0,80
-1,00
2006
2008
2010
2012
2014
2016
2018
2020
2022
historical mean
Historical mean + 2 SD
Historical mean - 2 SD
PT campaign
